# Graphene oxide decorated with gold enables efficient biophotovolatic cells incorporating photosystem I†

**DOI:** 10.1039/d1ra08908k

**Published:** 2022-03-22

**Authors:** Nahid Torabi, Sylvia Rousseva, Qi Chen, Ali Ashrafi, Ahmad Kermanpur, Ryan C. Chiechi

**Affiliations:** Stratingh Institute for Chemistry, University of Groningen Nijenborgh 4 9747 AG Groningen The Netherlands; Zernike Institute for Advanced Materials Nijenborgh 4 9747 AG Groningen The Netherlands; Department of Materials Engineering, Isfahan University of Technology Isfahan 84156-83111 Iran ahmad_k@iut.ac.ir; Department of Chemistry, North Carolina State University Raleigh North Carolina 27695-8204 USA rchiech@ncsu.edu

## Abstract

This paper describes the use of reduced graphene oxide decorated with gold nanoparticles as an efficient electron transfer layer for solid-state biophotovoltic cells containing photosystem I as the sole photo-active component. Together with polytyrosine–polyaniline as a hole transfer layer, this device architecture results in an open-circuit voltage of 0.3 V, a fill factor of 38% and a short-circuit current density of 5.6 mA cm^−2^ demonstrating good coupling between photosystem I and the electrodes. The best-performing device reached an external power conversion efficiency of 0.64%, the highest for any solid-state photosystem I-based photovoltaic device that has been reported to date. Our results demonstrate that the functionality of photosystem I in the non-natural environment of solid-state biophotovoltaic cells can be improved through the modification of electrodes with efficient charge-transfer layers. The combination of reduced graphene oxide with gold nanoparticles caused tailoring of the electronic structure and alignment of the energy levels while also increasing electrical conductivity. The decoration of graphene electrodes with gold nanoparticles is a generalizable approach for enhancing charge-transfer across interfaces, particularly when adjusting the levels of the active layer is not feasible, as is the case for photosystem I and other biological molecules.

## Abstract 2

This paper describes the use of reduced graphene oxide decorated with gold nanoparticles as an efficient electron transport layer for solid-state biophotovoltic cells containing photosystem I as the sole photo-active component.

## Graphical abstract

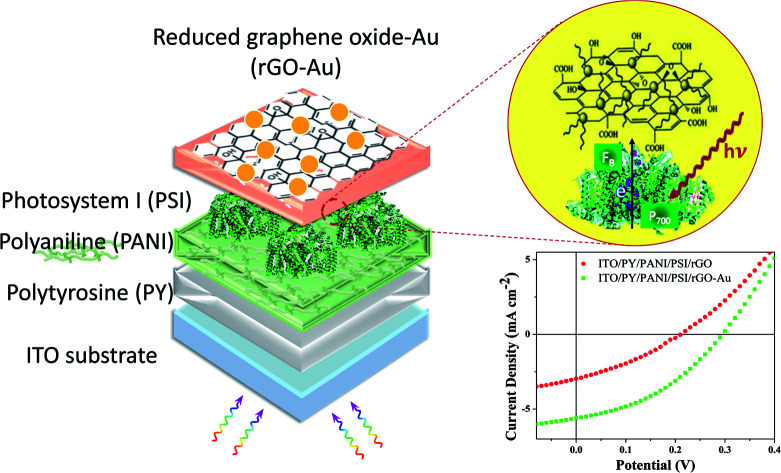

## 1 Introduction

Photosystem I (PSI) is a trimeric photoactive electron-transport protein complex that is found in thylakoid membranes and is a part of the photosynthetic apparatus of green plants, algae, and cyanobacteria.^1–4^ It is a light-harvesting element, and its ability to convert solar energy into spatially separated charges,^5^ combined with its ease of extraction and natural abundance^6,7^ makes it ideal for sustainable, biomimetic energy conversion applications. A series of processes in PSI transfer energy from light-harvesting pigments at the periphery into a multi-step electron transport chain beginning at the reaction center (P_700_)^8^ that generates a hole on P_700_ and an electron on the iron–sulfur cluster (F_B_–Fe_4_S_4_) acceptor site,^9^ which are at the termini of the chain, with a quantum efficiency near unity.^10^ The operating principle of PSI-based biophotovoltaic (BPV) cells involves capturing these electrons and holes as they are generated on opposite sides of each PSI complex following the absorption of a photon.

A major challenge to integrating PSI into BPV cells is that, during the extraction and isolation process of PSI, the PSI trimers must be removed from the thylakoid membrane, which destabilizes them and reduces their efficiency.^5^ Solid-state solar cells are comprised of multiple layers stacked on top of each other; thus, in BPV cells, PSI must be sandwiched between charge-transfer layers, and the selection of electron- and hole-transfer materials with the proper energy alignments plays a critical role in facilitating the transfer of charge to and from individual PSI complexes.^6,11^ To achieve continuous, optimal charge-transfer flux through these different layers and to maximize the efficiency of solid-state BPV cells, the PSI complexes need to be kept properly aligned with respect to the hole/electron injection and structurally intact.^12^ Several methods have been developed to achieve this, such as increasing light absorption by using thick multilayer films of PSI,^11,13^ increasing the surface area of the electrodes by using nanoporous and roughened substrates,^8,14,15^ wiring isolated PSI complexes with modified electrodes^16–18^ and using carbon-based electrodes.^1,7^ Recent investigations on the topic of performance improvements of BPV cells have focused on the selection of electrodes, mediators, and composite matrices with optimal properties.^13,19–21^

Increasing the surface-area of electrodes is a general strategy for increasing photocurrent in PSI-based BPV devices, in combination with conformal charge-transfer layers. Nanoporous films of tin-doped indium oxide (ITO) nanoparticles, microporous inverse-opal structures, needle-like polytyrosine (PY), and conductive polymers have been shown to increase the efficiency of BPV devices.^22–25^ Polyaniline (PANI) has been utilized as a conformal hole-transfer layer in BPVs due to its high electrical conductivity, charge mobility, and compatibility with PSI complexes.^26–30^ Nano-composites based on carbon allotropes such as graphene, graphene oxide (GO) and reduced graphene oxide (rGO) exhibit a similar combination of useful properties: high electrical conductivity, large surface area, mechanical flexibility, good carrier mobility, thermal conductivity and transparency in the visible range of the spectrum.^7,11,21,31–33^ These materials have been used as charge-transfer layers in silicon-based solar cells,^34–36^ dye-sensitized solar cells^37–39^ and other energy storage devices.^27,40–42^ In previous studies, when PSI was deposited onto GO surfaces, the hydroxy (–OH), carboxylic acid (–COOH), and epoxide groups present in GO make it particularly well suited for PSI-based BPV devices because they interact selectively with PSI to affect the orientation of the hole- and electron-injecting sides with respect to the charge-transfer layers and electrodes.^1,7^ These groups can also be used to tune the electric properties of GO by varying the oxygen quantity of the layers.^43,44^ Examples of successful strategies that utilize these properties in PSI-based BPV cells include coupling PSI on graphene electrodes modified with NHS-pyrene (*N*-hydroxysuccinimid) esters,^17^ immobilizing PSI on three-dimensional rGO electrodes modified with cytochrome c,^31^ directly conjugating PSI to graphene,^7^ incorporating oriented PSI in single-layer graphene flakes functionalized with Ni^2+^ nitrilotriacetic acid chelates (Ni-NTA), cytochrome *c*_553_,^45^ and depositing composite films of PSI with GO and rGO on p-doped silicon.^46^ All of these studies demonstrate significant progress in the maximizing of photocurrents in BPV devices. Lastly, due to the unique physicochemical properties, bio-inertness, and high catalytic activity,^12,47–49^ gold nanoparticles (AuNPs) can increase the rate of electron transfer and the electrical response of PV devices under illumination.^42^ Dispersions of GO and rGO with AuNPs are widely used in sensing applications,^41,50,51^ solar cells^33^ and to enhance photocatalytic H_2_ production.^43^ Here, we utilize the unique properties of these materials by decorating rGO with AuNPs to enhance the performance of PSI-based BPV cells. We combine the aforementioned approaches to construct cells in which PSI complexes are self-assembled on a hole-transfer layer of PY-PANI on ITO electrodes and covered with an electron-transfer layer of rGO decorated with AuNPs (rGO–Au). Using this combination of materials, we show the highest external power conversion efficiency (*η*) for PSI-based BPVs devices reported to date.

## 2 Results and discussion

### 2.1 Device design

**Fig. 1:**
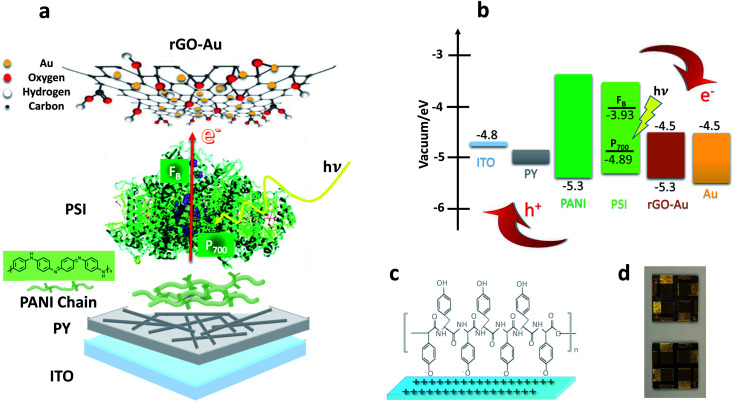
(a) Solid-state PSI-based photovoltaic cell with labeled components. (b) Energy levels of each layer and charge transfer pathways to external electrodes of biophotovoltaic cell. (c) Assembly of polytyrosine on positive ITO surface. (d) A photograph of completed BPV devices (the energy level data are given elsewhere.^23,53–57^).

The overall device structure, energy levels, and associated electron and hole transfer pathways of our BPV cells are schematically depicted in Fig. 1 where ITO serves as the cathode, PY-PANI as the hole transfer layer (HTL), PSI as an active layer, rGO–Au as an electron transfer layer (ETL) and evaporated gold serves as the anode. Solid-state BPV cell fabrication begins with cleaning pre-patterned ITO glass in several steps; first, substrates were cleaned using soap and DI water (DIW) under sonication. Then, they were cleaned by sonication in acetone and in the last step sonication in isopropanol. Finally, they were dried in a stream of N_2_ and then activated in an O_2_ plasma-cleaner for 3 min before dipping them into a PY solution (pH 7.4) at 5 °C. After 3 d, the substrates were removed from the PY solution and gently immersed in deionized water to remove any unbound PY colloids. During this process, the ITO is coated in a layer of PY due to the electrostatic attraction between the activated ITO and PY; we presume the oxygen plasma treatment leaves a net positive charge on the ITO surface,^52^ because PY, which bears acidic phenol moieties that are readily deprotonated, is net-negatively charged, as is depicted schematically in Fig. 1c. A PANI solution was then spin-coated onto the PY layer at 1000 rpm (with 1000 rpm s^−1^ acceleration) for 30 s. The PSI active layer was deposited by drop-casting an aqueous solution (40 μL at 0.8 μM), which was allowed to stand on the bench-top in the cleanroom environment at a temperature of 20 °C for 30 min. After 30 min, the samples were then dried completely *in vacuo* at room temperature for 45 min. In the next step of device fabrication, rGO–Au (≈1 mg mL^−1^, see Experimental) was deposited by spin coating at 500 rpm (with 350 rpm s^−1^ acceleration) for 120 s, then at 1000 rpm (with 500 rpm s^−1^ acceleration) for 30 s before being gently dried in a stream of N_2_. Finally, the 60 nm-thick Au anode was thermally deposited directly onto the rGO–Au layer under high vacuum (3 × 10^−8^ mbar). The active area was determined by measuring the overlap of the Au anode and the pre-patterned ITO cathode.

### 2.2 Device performance

**Fig. 2:**
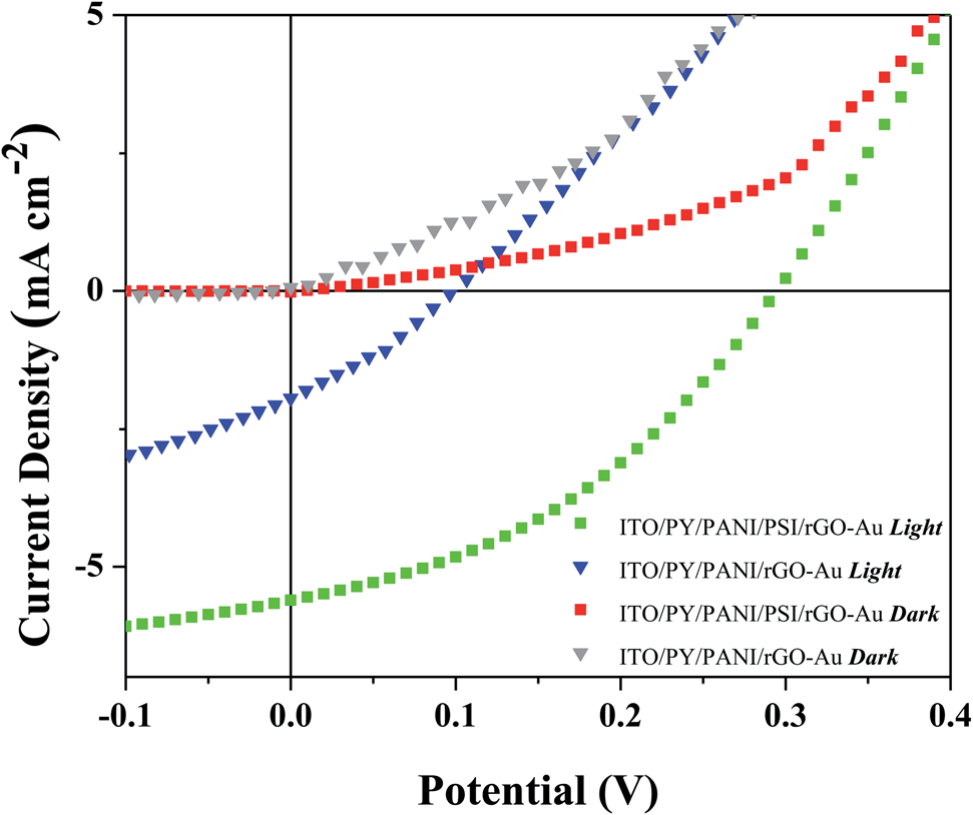
*J*–*V* plots of BPV cells with and without PSI in dark and under simulated sunlight with an intensity of 1000 W m^−2^.

As depicted in Fig. 1, the expected three-step mechanism is: (1) the reaction center (P_700_) of each PSI complex absorbs light; (2) electrons are shuttled to the (F_B_–Fe_4_S_4_) acceptor site, while holes remain localized at the P_700_ donor site; and (3) the holes travel through the PY-PANI HTL to the ITO cathode and electrons travel through the rGO–Au ETL to the Au anode. The resulting current *J*–*V* characteristics of BPV cells in the dark and illuminated with AM1.5G simulated sunlight in the presence and absence of the PSI active layer is shown in Fig. 2, and the relevant photovoltaic parameters are summarized in Table S1.† These results demonstrate that, the performance metrics of open-circuit voltage (*V*_OC_), short-circuit current density (*J*_SC_), fill factor (FF) and external power conversion efficiency *η* are maximized in cells with PSI active layers under illumination, demonstrating the generation and collection of photo-generated charge by PSI complexes. Under illumination devices with the configuration ITO/PY/PANI/PSI/rGO–Au/Au yielded a *V*_OC_ of 0.3 V and *J*_SC_ of 5.6 mA cm^−2^, while in the absence of PSI, devices with the configuration ITO/PY/PANI/rGO–Au/Au yielded *V*_OC_ and *J*_SC_ that were approximately threefold smaller. Furthermore, *η* increases tenfold when PSI is included in the device configuration.

**Fig. 3:**
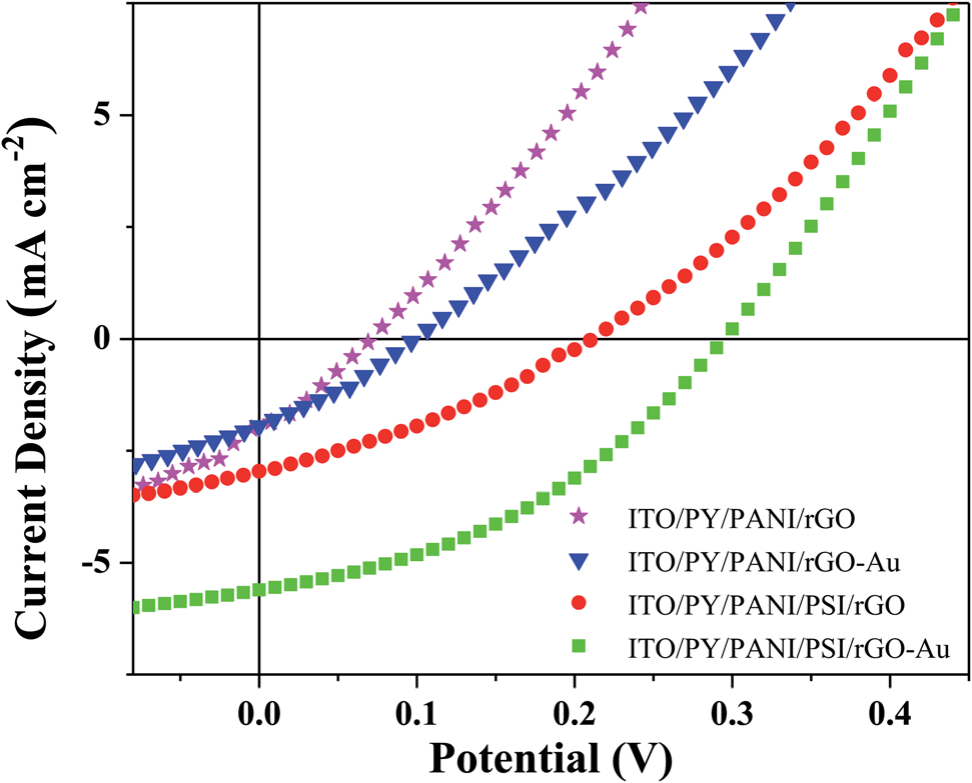
*J*–*V* curves of four different photovoltaic cells fabricated with rGO–Au and rGO layer in the presence and absence of PSI under AM1.5G illumination. BPV cells fabricated of ITO/PY/PANI/PSI/rGO–Au (green), ITO/PY/PANI/PSI/rGO (blue), ITO/PY/PANI/rGO–Au (orange), and ITO/PY/PANI/rGO (magenta).

To elucidate the role of the AuNPs, BPV cells were prepared with rGO electron-transfer layers lacking AuNPs while keeping the rest of the device configuration constant. The comparison of *J*–*V* measurements of BPV cells with or without AuNPs layers in the presence and absence of PSI is presented in Fig. 3; comparison of the device perfomance, also summarized in Table 1, demonstrates that *V*_OC_ and *J*_SC_ of 0.21 V and 2.96 mA cm^−2^ obtained for the BPV cells without AuNPs, result in a significantly lower device performance and a threefold decrease in PCE relative to the devices with AuNPs. These results clearly demonstrate that surface functionalization of 2D layers of graphene by integration of low-dimensional AuNPs is an effective method to improve the performance of PSI-based photovoltaic devices which increase photochemical activity through plasmonic coupling and retain the structural integrity and charge-carrier mobility at the interface.^42,58^ Specifically, the surface functionalization of rGO with AuNPs adjusts the work function^33^ and lowers contact resistance, increasing *J*_SC_ and FF by facilitating the extraction of charges from the photo-excited PSI complexes before they recombine.^59,60^ Meanwhile, the increased work function gives rise to an improved *V*_OC_ in the BPV devices. The alignment of the energy levels of the transfer layers with PSI (Fig. 1) is particularly important for reducing recombination because it ensures that there is no energetic barrier to the extraction of charges from the PSI complexes. The evaluation of current density *versus* voltage for BPV devices fabricated using ITO/PY/PANI/PSI/rGO–Au/Au was measured during 10 d (Fig. S4†) which shows that, as the PSI layer ages, there are fewer intact PSI complexes contributing to the photovoltaic effect and the total performance of BPV devices decreases.

**Table 1:** Performance parameters of four different photovoltaic cells fabricated of rGO–Au and rGO layer as electron transfer layer in the presence and absence of PSI. The errors were obtained from the standard deviation of multiple test samples. The abbreviations used are tin-doped indium oxide (ITO), polytyrosine (PY), polyaniline (PANI), reduced graphene oxide (rGO), gold (Au), photosystem I (PSI), open-circuit voltage (*V*_OC_), short-circuit current density (*J*_SC_), fill factor (FF), and external power conversion efficiency(*η*)

Devices	*V*_OC_(V)	*J*_SC_ (mA cm^−2^)	FF	*η* (%)
ITO/PY/PANI/PSI/rGO–Au/Au	0.3 ± 0.02	5.6 ± 0.28	0.38 ± 0.06	0.64 ± 0.03
ITO/PY/PANI/PSI/rGO/Au	0.21 ± 0.01	2.96 ± 0.37	0.32 ± 0.09	0.2 ± 0.05
ITO/PY/PANI/rGO–Au/Au	0.1 ± 0.01	1.95 ± 0.14	0.3 ± 0.12	0.06 ± 0.01
ITO/PY/PANI/rGO/Au	0.07 ± 0.03	2 ± 0.41	0.29 ± 0.07	0.04 ± 0.01

**Fig. 4:**
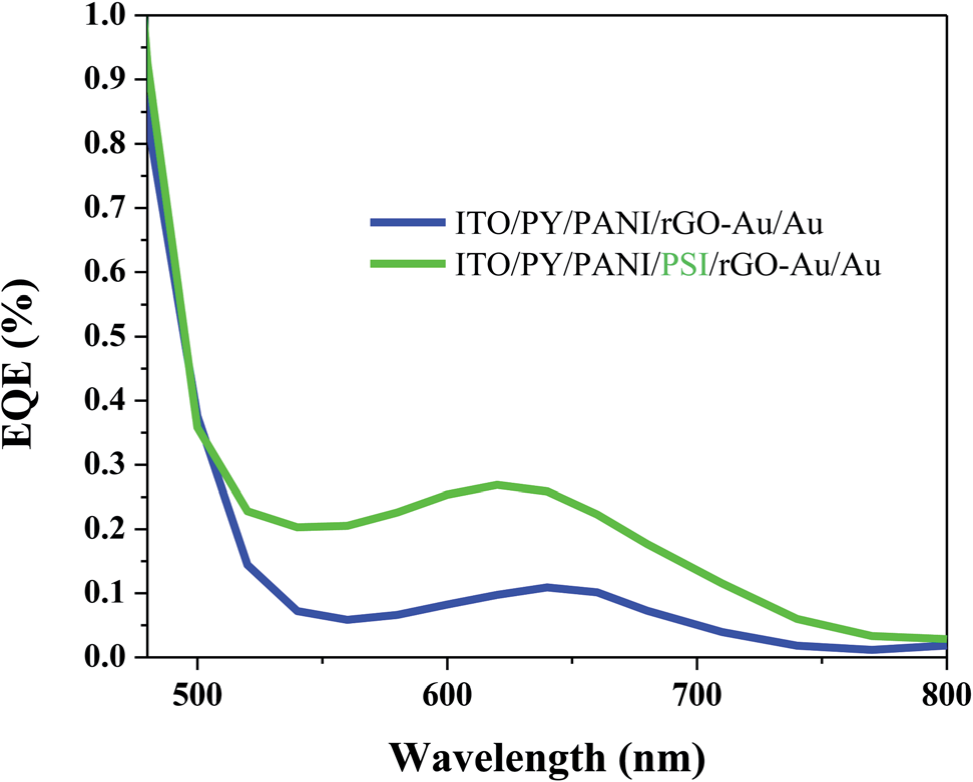
External quantum efficiency (EQE) of fabricated BPV cells with and without PSI as active layer between PANI and rGO–Au charge transfer layers.

EQE spectra of BPV cells with the configuration ITO/PY/PANI/PSI/rGO–Au/Au and ITO/PY/PANI/rGO–Au/Au (Fig. 4) were measured to evaluate the photovoltaic performance, further confirming the active role of the PSI layer. In BPV cells with the configuration ITO/PY/PANI/PSI/rGO–Au/Au, there is a peak between 600 nm to 700 nm and the magnitude of the EQE spectrum is significantly enhanced in the same spectral region as the absorption of PSI (Fig. S1†). A reference cell with the configuration ITO/PY/PANI/rGO–Au/Au (*i.e.*, lacking PSI) gave a significantly smaller response in this spectral region, with an EQE response that can be ascribed to the absorption of PANI (see Fig. S2†). The integrated current-densities estimated from the EQE spectra are also in good agreement with the *J*_SC_ values calculated from the *J*–*V* curves.

To contextualize our results, Table 2 gives an overview of recent advances in the performance of solid-state and electrolyte-based BPV cells that use active layers of PSI, which have been steadily improving in photocurrents and photovoltages over the past ten years. While there are many factors that determine the performance of BPV cells (*e.g.*, electrode materials, surface-to-volume ratio, electrolytes, and immobilization of PSI) the selection of transfer layers is vital because the energy levels of PSI are fixed and the internal level-alignment both affects device performance and mitigates damage from internal processes. As can be seen from Table 2, improvements in one or two of the performance parameters does not translate into high efficiencies. In this work, the deposition of rGO–Au onto PSI complexes yields a comparatively good *V*_OC_, which is mainly a function of energy alignment. The PY/PANI interface facilitates good density and alignment (with respect to the injection of holes and electrons) of PSI, leading to good *J*_SC_. Both of these factors—transfer layers and alignment—contribute to FF, which is strongly influenced by the relative rate of recombination. While neither *J*_SC_, *V*_OC_ nor FF are the highest reported, the combination of the three lead to an optimized efficiency of *η* = 0.64 ± 0.03, which is the highest reported to date. Ultimately, *η* is the most important metric for BPV device performance, as it captures the efficiency with which photon energy is converted into electrical energy.

**Table 2:** Performance parameters of different PSI-based photovoltaic devices from the recent literature with different charge transfer materials. The abbreviations used are fluorine-doped tin oxide (FTO), aminoethanethiol (AET), layer by layer photosystem I (LBL PSI), perylene di-imide (PTCDI), poly[2-methoxy-5-(2′-ethylhexyloxy)-1,4-phenylene vinylene] (MEH-PPV), (6,6)-Phenyl C61 butyric acid methyl ester (PCBM), poly[bis(4-phenyl)(2,4,6-trimethylphenyl)amine] (PTAA), poly(*p*-xylylviologen) (P_*x*_*V*), poly(3,4-ethylenedioxythiophene)polystyrene sulfonate (PEDOT:PSS), sodium 3-mercapto-1-propanesulfonate (MPS), phenyl-C_61_-butyric acid (PCBA)

Device configuration	Type of BPV cells		*V*_OC_ (V)	*J*_SC_ (mA cm^−2^)	FF	*η* (%)	Reference
	Solid-state	Electrolyte					
FTO/TiO_2_/PSI	—	✓	0.5	0.362	0.71	0.13	61
ITO/PSI/MEH-PPV:PCBM/MoO_3_/Al	✓	—	0.34	0.305	0.4	0.041	62
FTO/TiO_2_/PANI-PSI/Ag	✓	—	0.299	0.072	0.42	0.0091	30
Au/PANI-PSI	—	✓	—	0.0057	—	0.005	27
FTO/TiO_2_/PSI	—	✓	0.59	1.3	0.62	0.47	63
P-doped silicon/PSI/ZnO/ITO	✓	—	0.214	0.127	0.28	0.0077	6
FTO/PEDOT:PSS/PSI/LiF/Al	✓	—	0.25	0.96	0.31	0.069	64
ITO/PY/PSI/C_60_/Au	✓	—	0.36	3.47	0.33	0.517	23
PAni/PSI/TiO_2_/SnO_2_	✓	—	0.299	0.072	0.42	0.0091	65
ITO/P_*x*_V/PSI/P-Si	✓	—	0.25	0.027	—	0.002	66
FTO/TiO_2_/PTCDI	—	✓	0.43	0.43	0.62	0.12	67
Au/AET/PSI-PEDOT:PSS LBL	—	✓	—	0.00041	—	—	68
Au/MPS/PSI	—	✓	0.5	0.18	0.15	0025	69
FTO/TiO_2_/PSI	—	✓	0.443	0.175	0.43	0.042	70
Au/PCBA/PSI	—	✓	0.59	0.12602	0.31	0.0043	71
ITO/PY/PANI/PSI/rGO–Au/Au	✓	—	0.3	5.6	0.38	0.64	This study

## 3 Conclusions

The active components of biological light-harvesting systems, such as PSI, are a naturally abundant and renewable resource that can be developed into affordable, safe, and efficient solar cells. But biological light-harvesting converts photon energy into chemical energy. Finding the right combination of materials for co-opting these systems to produce electrical energy, especially in solid-state devices, remains a challenge. Our results demonstrate that using rGO–Au as an ETL facilitates the extraction of photo-generated electrons without sacrificing other parts of the power conversion process. This remarkable enhancement affords PSI-based PV cell with the highest reported power conversion efficiency to date, *η* = 0.64%. This work establishes a foundation for utilizing the unique properties of graphene-based materials decorated with metal nanoparticles in future BPV devices. Further studies will optimize the electrical interaction between carbon-based materials and PSI for proper electron transfer and improved photoresponsivity as well as long-term stability, which is an important step on the way to potential technological applications.

## 4 Experimental

### 4.1 Materials

ITO, l-tyrosine (Sigma-Aldrich, 99%), polyaniline (emerladine salt, average *M*_w_ > 15 000, Sigma-Aldrich), tetrachloroauric(iii) acid (HAuCl_4_, Sigma-Aldrich, 99.9%wt), l-ascorbic acid (Sigma-Aldrich, 99%), graphite powder, phosphoric acid (H_3_PO_4_), sulfuric acid (H_2_SO_4_), potassium permanganate (KMnO_4_), hydrochloric acid (HCl), hydrogen peroxide (H_2_O_2_) were purchased and used as recieved. For all experiments, DIW was used.

### 4.2 Extraction and isolation of PSI

PSI was extracted from cyanobacterium *Thermosynechococcus elongatus* as described previously.^69^ Briefly, thermophilic cyanobacterium *Thermosynechococcus elongatus* BP-1 was grown in BG11 medium, and then cells were collected by centrifugation under controlled conditions. Afterward, by re-suspending cells in a buffer and after several centrifuge cycles, the thylakoid membranes were broken open.^72^ The purified PSI complexes were collected by using an anion exchange column chromatography. Chlorophyll a concentration of the resulting PSI solution was determined by the methods of Porra^73^ and Baba *et al.*^74^

### 4.3 Synthesis of reduced graphene oxide decorated with gold

The preparation of graphene oxide (15 mg mL^−1^) was performed by Hammers^75^ method through the oxidation of graphite. The decoration of rGO by AuNPs was achieved using the procedure previously described.^76^ Briefly, 0.1 g of GO was dispersed in 100 mL of DI water (1 mg mL^−1^). To this aqueous solution, 20 mL of ascorbic acid solution (20 mM) was added, and GO reduced well on a magnetic stirrer at 90 °C for 12 h. Then, 40 mL of aqueous solution of HAuCl_4_ (1 mg mL^−1^) was slowly added to the prepared suspension on a magnetic stirrer. The resulting mixture was then continually stirred for 24 h under reflux conditions.

### 4.4 Current–voltage (J–V) characterization

The current–voltage curves of solid-state BPV cells were measured in the glove box by means of a Keithley 2400 source meter in the dark and under simulated AM1.5G white light illumination in an inert atmosphere. The intensity of the white light was calibrated using a mono-silicon reference cell for one sunlight intensity of 1000 W m^−2^. All cells were measured at room temperature (295 K), and the temperature maintained by means of a liquid N_2_ bath. To define the illuminated cell area, a black shadow mask with an aperture area was used. Keithley swept the voltage from 0.5 to −0.2 for the reverse scan and from −0.2 to 0.5 for the forward scan with a step size of 0.01 V. The time delay of the Keithley 2400 source meter was 0.01 s and the total time between the voltage range of −0.2 to 0.5 was 0.71 s.

### 4.5 External quantum efficiency measurements

For measurements of external quantum efficiency, a home-built setup was used. The light from a xenon lamp (Newport, operating power 230 W) was guided through a set of three filter wheels (Spectral Products, AB304-T) which allowed measurements in the spectral range 400–1400 nm at 20 nm intervals below 680 nm and 30 nm intervals above 680 nm. After the filter wheels, the light was focused using a lens, passed through an aperture and a chopper (Stanford Research instruments, 78 Hz), and then further focused with a series of lenses to obtain a spot size smaller than the active area of the device. The sample was held in place in an air-tight sample holder, and the obtained photocurrent was measured using a lock-in amplifier (Stanford Research Systems, Model SR830 DSP Lock-In Amplifier). All measurements were carried out in a dark room, and prior to measurement of the device, the photon flux was calibrated using a set of Newport optical power detectors (Newport Model 818-SL and Model 818-IR).

### 4.6 UV-Vis absorption

UV-Vis absorption spectra were measured by JENWAY 6715 UV-Vis spectrophotometer at room temperature between 200 nm and 800 nm.

## Conflicts of interest

There are no conflicts to declare.

## Supplementary Material

RA-012-D1RA08908K-s001
