# Supplementary material for: Graphene oxide decorated with gold enables efficient biophotovolatic cells incorporating photosystem I
Source: RSC Adv. 2022 Mar 22;12(14):8783–91. doi: 10.1039/d1ra08908k (PMC8984948; doi:10.1039/d1ra08908k)
Supplement: RA-012-D1RA08908K-s001 [file RA-012-D1RA08908K-s001.pdf]

Electronic Supplementary Material (ESI) for RSC Advances.
This journal is © The Royal Society of Chemistry 2022

Supporting Information for: Graphene Oxide Decorated with Gold Enables Efficient Biophotovolatic Cells Incorporating Photosystem I

Nahid Torabi[1,2,3], Sylvia Rousseva[1,2], Qi Chen[2], Ali Ashrafi[3], Ahmad Kermanpur*[3], and Ryan C. Chiechi*[1,2,4]

[1]*Stratingh Institute for Chemistry, University of Groningen, Nijenborgh 4, 9747 AG Groningen, the Netherlands*
[2]*Zernike Institute for Advanced Materials, Nijenborgh 4, 9747 AG Groningen, the Netherlands*
[3]*Department of Materials Engineering, Isfahan University of Technology, Isfahan 84156-83111, Iran*
[4]*Department of Chemistry, North Carolina State University, Raleigh, North Carolina 27695-8204, United States*
**e-mail: ahmad_k@iut.ac.ir*
**e-mail: rchiech@ncsu.edu*

Contents

1 Photovoltaic parameters of fabricated BPV cells with and without PSI under illumination. **S-2**

2 Ultraviolet-visible (UV-vis) spectroscopy of different materials used in BPV devices **S-3**

3 Evaluation of current density of BPV devices during 10 days **S-5**

4 Optical and Scanning Electron Microscopy (SEM) micrographs **S-6**

1 Photovoltaic parameters of fabricated BPV cells with and without PSI under illumination.

Table S1: Photovoltaic parameters of fabricated BPV cells with and without PSI under illumination. The errors were obtained from the standard deviation of multiple test samples.

Devices	V_{OC} (V)	J_{SC} (mA cm^{-2})	FF	η (%)
**ITO/PY/PANI/PSI/rGO-Au/Au**	0.3 ± 0.02	5.6 ± 0.28	0.38 ± 0.06	0.64 ± 0.03
**ITO/PY/PANI/rGO-Au/Au**	0.1 ± 0.01	1.95 ± 0.14	0.3 ± 0.12	0.06 ± 0.01

2 Ultraviolet-visible (UV-vis) spectroscopy of different materials used in BPV devices

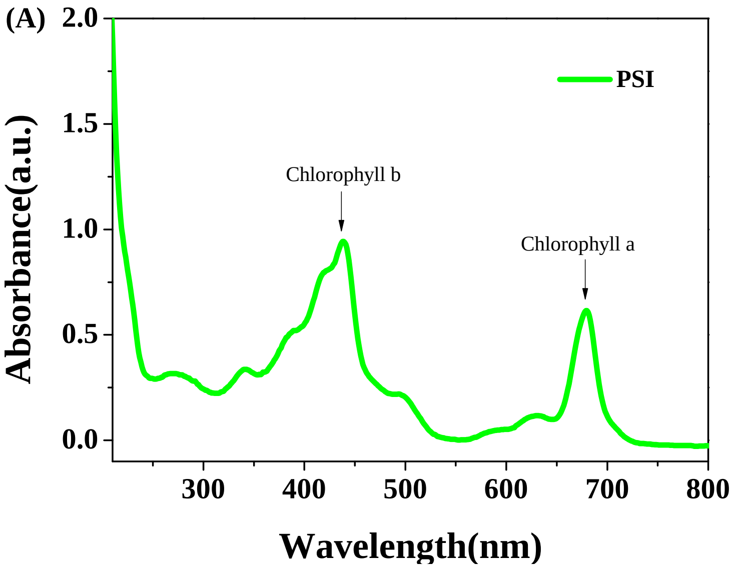

Figure S1: Absorption spectrum of PSI with two characteristic absorption peaks at 430 nm and 670 nm

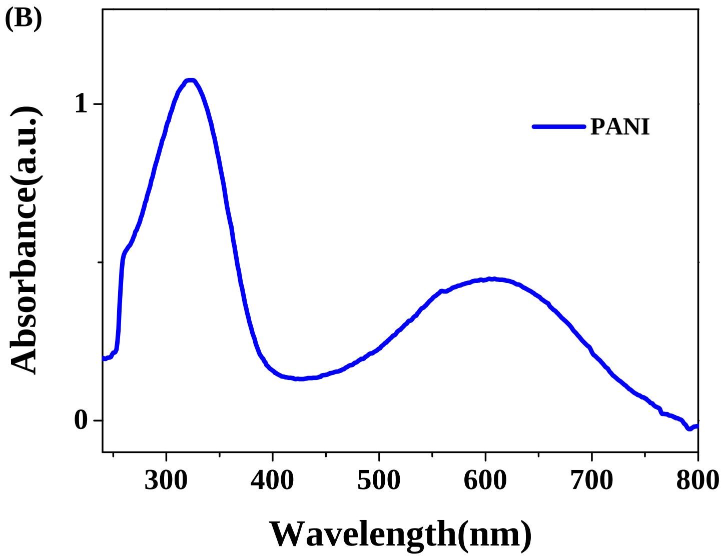

Figure S2: UV-Vis. spectra of conducting polymer polyaniline (PANI).

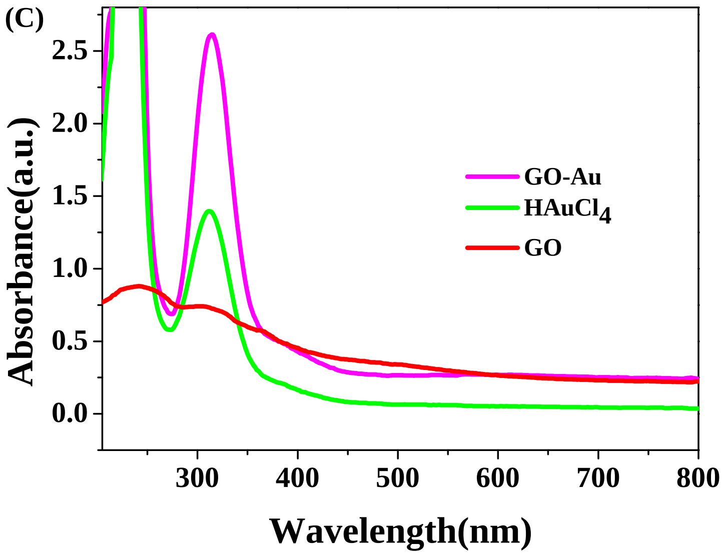

Figure S3: UV-Vis. Spectra of graphene oxide (GO), HAuCl4 and reduced graphene oxide decorated with Au (rGO-Au).

3 Evaluation of current density of BPV devices during 10 days

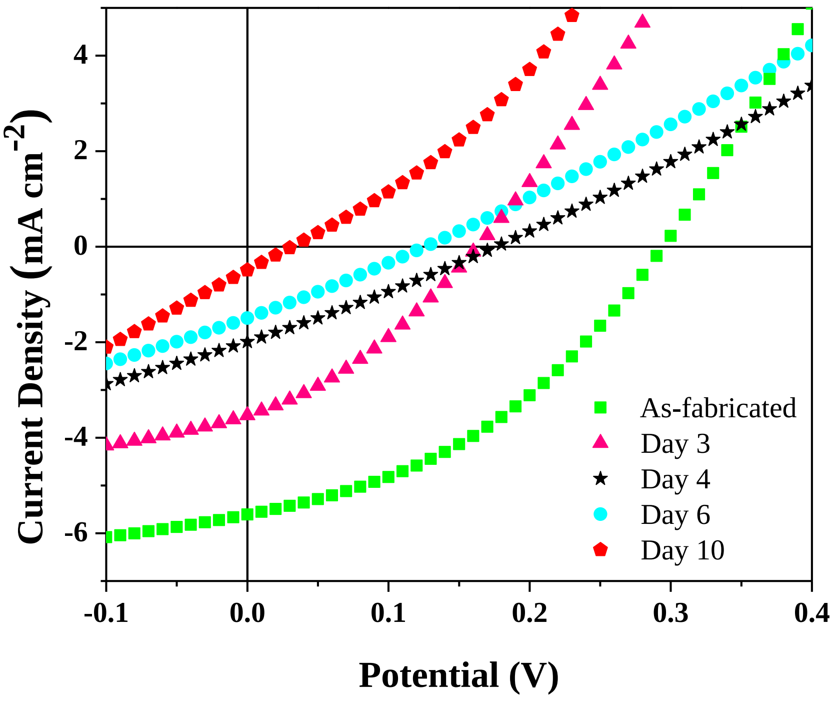

Figure S4: Evaluation of current density of BPV devices fabricated using ITO/PY/PANI/PSI/rGO-Au/Au during 10 days.

4 Optical and Scanning Electron Microscopy (SEM) micrographs

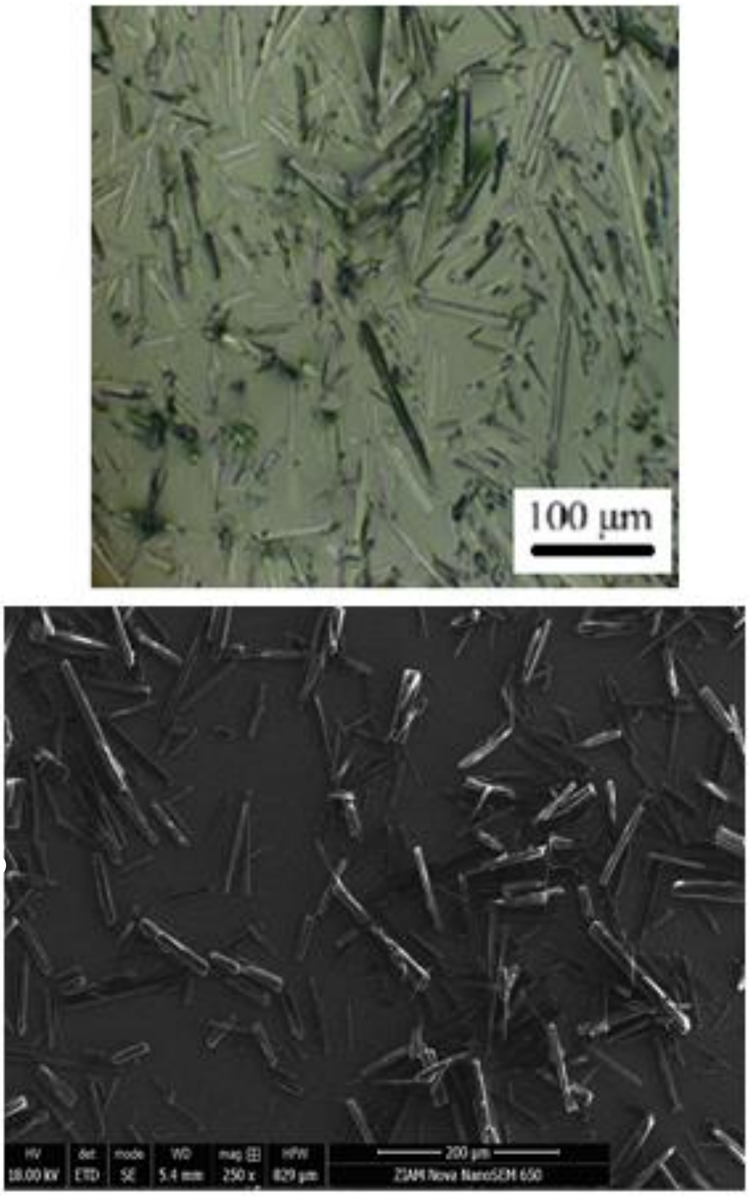

Figure S5: A) Optical micrograph and B) SEM micrograph of PSI complexes on PY layers.
